# CaMKIIδ-dependent dysregulation of atrial Na^+^ homeostasis promotes pro-arrhythmic activity in an obstructive sleep apnea mouse model

**DOI:** 10.3389/fphar.2024.1411822

**Published:** 2024-06-20

**Authors:** Philipp Hegner, Florian Ofner, Benedikt Schaner, Mathias Gugg, Maximilian Trum, Anna-Maria Lauerer, Lars Siegfried Maier, Michael Arzt, Simon Lebek, Stefan Wagner

**Affiliations:** ^1^ Department of Internal Medicine II, University Hospital Regensburg, Regensburg, Germany; ^2^ Department of Neurology and Clinical Neurophysiology, University Hospital Augsburg, Augsburg, Germany

**Keywords:** sleep-disordered breathing, reactive oxygen species, CaMKIIδ, Na^+^ homeostasis, cardiac arrhythmias, obstructive sleep apnea

## Abstract

**Background:**

Obstructive sleep apnea (OSA) has been linked to various pathologies, including arrhythmias such as atrial fibrillation. Specific treatment options for OSA are mainly limited to symptomatic approaches. We previously showed that increased production of reactive oxygen species (ROS) stimulates late sodium current through the voltage-dependent Na^+^ channels via Ca^2+^/calmodulin-dependent protein kinase IIδ (CaMKIIδ), thereby increasing the propensity for arrhythmias. However, the impact on atrial intracellular Na^+^ homeostasis has never been demonstrated. Moreover, the patients often exhibit a broad range of comorbidities, making it difficult to ascertain the effects of OSA alone.

**Objective:**

We analyzed the effects of OSA on ROS production, cytosolic Na^+^ level, and rate of spontaneous arrhythmia in atrial cardiomyocytes isolated from an OSA mouse model free from comorbidities.

**Methods:**

OSA was induced in C57BL/6 wild-type and CaMKIIδ-knockout mice by polytetrafluorethylene (PTFE) injection into the tongue. After 8 weeks, their atrial cardiomyocytes were analyzed for cytosolic and mitochondrial ROS production via laser-scanning confocal microscopy. Quantifications of the cytosolic Na^+^ concentration and arrhythmia were performed by epifluorescence microscopy.

**Results:**

PTFE treatment resulted in increased cytosolic and mitochondrial ROS production. Importantly, the cytosolic Na^+^ concentration was dramatically increased at various stimulation frequencies in the PTFE-treated mice, while the CaMKIIδ-knockout mice were protected. Accordingly, the rate of spontaneous Ca^2+^ release events increased in the wild-type PTFE mice while being impeded in the CaMKIIδ-knockout mice.

**Conclusion:**

Atrial Na^+^ concentration and propensity for spontaneous Ca^2+^ release events were higher in an OSA mouse model in a CaMKIIδ-dependent manner, which could have therapeutic implications.

## 1 Introduction

Over the past few decades, sleep-disordered breathing (SDB) has emerged as a highly prevalent disease that currently affects about one billion patients worldwide (Benjafield et al., 2019). SDB is frequently associated with various cardiovascular disorders, such as hypertension (Pengo et al., 2020), heart failure with reduced or preserved ejection fractions (HFrEF/HFpEF) (Arzt et al., 2016; Lebek et al., 2021; Wester et al., 2023; Hegner et al., 2024), and arrhythmias like atrial fibrillation (Gami et al., 2004; Hegner et al., 2021a; Hegner et al., 2021b; Mehra et al., 2022), which may lead to subsequent strokes (Arzt et al., 2005). The interactions between SDB and these cardiovascular disorders can substantially contribute to patient morbidity and mortality while also posing economic challenges (Gami et al., 2004; Arzt et al., 2005; Arzt et al., 2016; Benjafield et al., 2019; Pengo et al., 2020; Lebek et al., 2021; Mehra et al., 2022; Wester et al., 2023). The current therapeutic strategies for SDB are mainly based on lifestyle interventions (e.g., weight loss, reduced alcohol intake, sports, and exercise) and continuous positive airway pressure (CPAP) therapy (Aurora et al., 2012; Randerath et al., 2017; Patil et al., 2019). However, patient compliance with these measures are often quite low, and adaptive servo-ventilation therapy has even been shown to increase mortality in HFrEF patients with central sleep apnea (Cowie et al., 2015; McEvoy et al., 2016). Thus, new and advanced therapeutic strategies are urgently needed for patients with SDB, which in turn requires detailed understanding of the pathological mechanisms involved.

We previously found increased production levels of reactive oxygen species (ROS) in human atrial biopsies of patients with SDB (Lebek et al., 2020b). This increase was shown to result in increased Ca^2+^/calmodulin-dependent protein kinase II (CaMKII) activation and enhanced CaMKII-dependent late Na^+^ current in the biopsies of patients with SDB (Lebek et al., 2020b; Lebek et al., 2022). Notably, the enhanced late Na^+^ current is an important trigger for early afterdepolarizations (EADs) and subsequent arrhythmias (Wagner et al., 2006; Sossalla et al., 2010; Glynn et al., 2015; Lebek et al., 2020b; Lebek et al., 2022). Indeed, we demonstrated an increased frequency of multicellular arrhythmias in the isolated trabeculae of patients with SDB that could be blocked with CaMKII inhibition as well as late Na^+^ current inhibition (Lebek et al., 2020b; Lebek et al., 2022). However, these studies were limited by patient heterogeneity and their various comorbidities that impacted myocardial Na^+^ homeostasis (Lebek et al., 2020b; Lebek et al., 2022). It is also unclear whether myocardial Na^+^ concentration is actually affected by the altered Na^+^ currents in SDB. Recently, we demonstrated for the first time that intracellular Na^+^ entry and Na^+^ concentration were higher in the atrial myocytes of patients with heart failure and preserved ejection fraction—conditions in which SDB is very common (Trum et al., 2024).

Therefore, we developed a mouse model of obstructive sleep apnea (OSA) by injecting polytetrafluorethylene (PTFE) into the murine tongue (Lebek et al., 2020a; Hegner et al., 2023); these mice developed diastolic and mild systolic left-ventricular dysfunctions after 8 weeks (Lebek et al., 2020a; Hegner et al., 2023). Importantly, this approach allows analysis of OSA mice without the confounding comorbidities that are frequently exhibited by patients. PTFE is an inert substance that permanently increases the murine tongue volume, thereby leading to increased frequency of apneas, inspiratory flow limitations (hypopneas), and subsequent hypoxemia (Lebek et al., 2020a; Hegner et al., 2023). Notably, these OSA events occur spontaneously in PTFE-injected mice and preferentially during the murine sleeping period, making this mouse model a suitable tool for investigating OSA-dependent effects in the absence of any potentially confounding comorbidities (Lebek et al., 2020a; Hegner et al., 2023). The objective of the current work was to explore whether atrial ROS production increased in the OSA mice that could subsequently lead to CaMKIIδ-dependent pro-arrhythmic dysregulation of atrial Na^+^ homeostasis.

## 2 Materials and methods

All experiments involving mice were in compliance with the directive 2010/63/EU of the European Parliament, Guide for the Care and Use of Laboratory Animals published by the US National Institutes of Health (NIH Publication No. 85–23, revised 1985), and local institutional guidelines. The government of Unterfranken, Bavaria, Germany also approved the animal protocol for this study (protocol number: 55.2-2532-2-512).

### 2.1 OSA induction by PTFE injection

**FIGURE 1:**
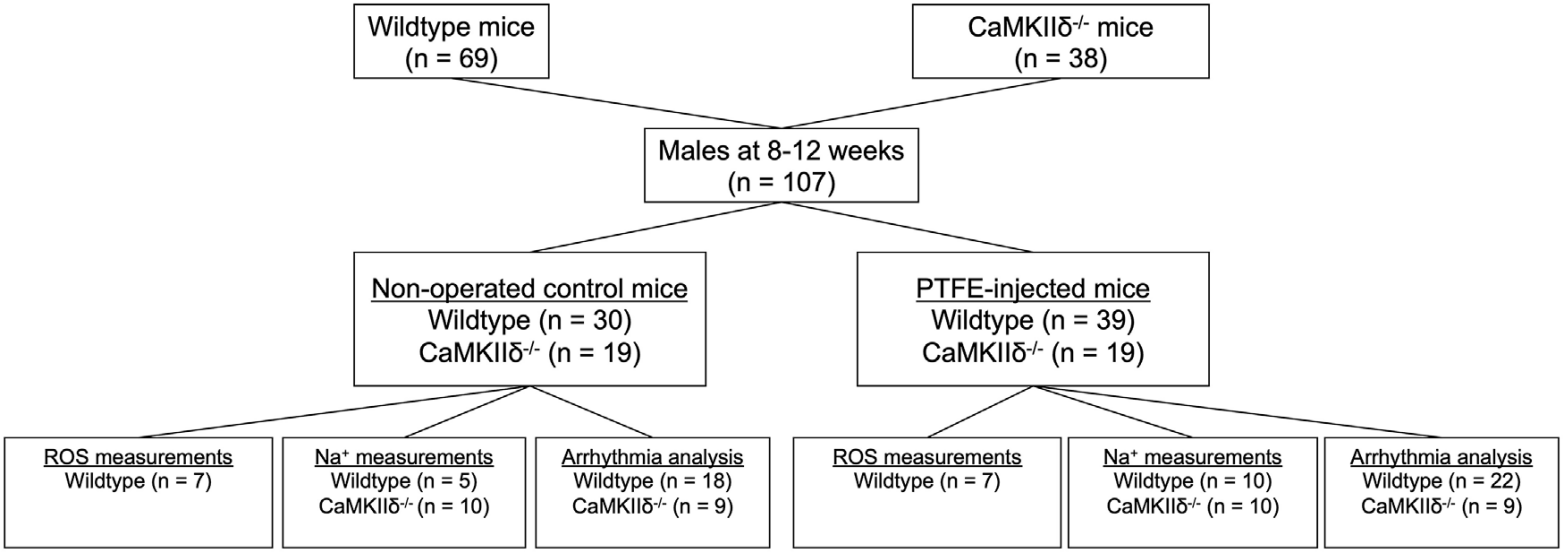
Experimental study flowchart.

OSA was induced in the study mice as described previously (Lebek et al., 2020a; Hegner et al., 2023). CaMKIIδ knockout (^−/−^) and C57BL/6 wild-type mice were randomly assigned to either the control (CTRL) or OSA induction by PTFE injection (PTFE) groups (Figure 1). The PTFE (35 μm particle size, Sigma-Aldrich) was injected into the tongues of the male mice at the age of 8–12 weeks (Lebek et al., 2020a). For optimal analgesia, the mice were treated with buprenorphine (0.1 mg/kg bodyweight intraperitoneal) 1 h before PTFE injection. Anesthesia was established using intraperitoneal injections of fentanyl (0.05 mg/kg bodyweight), medetomidine (0.5 mg/kg), and midazolam (5 mg/kg). Thereafter, the mice were placed on a heating plate in the supine position. The anesthesia was continuously monitored by recording the respiration and ECG, and the body temperature was monitored using a rectal probe. In total, 100 μL of diluted PTFE (50% w/v in glycerol, Sigma-Aldrich) was injected into multiple sites at the base of the tongue using a 27-gauge cannula. Ultrasound imaging was used to confirm successful PTFE injection into the tongue (Vevo3100 system, VisualSonics). Once the procedure was completed, the anesthesia was reversed using intraperitoneal injections of atipamezole (2.5 mg/kg), flumazenil (0.5 mg/kg), and buprenorphine (0.1 mg/kg bodyweight). The surgeries were performed by an experienced investigator who was blinded to the genotype of the mice. To reduce the stress on the animals, we refrained from revalidating the OSA severity resulting from PTFE injection as this was previously investigated in detail (Lebek et al., 2020a).

### 2.2 Isolation of atrial cardiomyocytes

The mouse atrial cardiomyocytes were isolated as described previously (Hegner et al., 2023). In brief, the explanted hearts were mounted on a Langendorff perfusion apparatus and retrogradely perfused with 113 mmol/L of NaCl, 4.7 mmol/L of KCl, 0.6 mmol/L of KH_2_PO_4_, 0.6 mmol/L of Na_2_HPO_4_ × 2 mmol/L of H_2_O, 1.2 mmol/L of MgSO_4_ × 7 mmol/L of H_2_O, 12 mmol/L of NaHCO_3_, 10 mmol/L of KHCO_3_, 10 mmol/L of HEPES, 30 mmol/L of taurine, 10 mmol/L of 2,3-butanedione monoxime, and 5.5 mmol/L of glucose for 4 min at 37°C (pH 7.4). Next, trypsin 0.6%, 7.5 mg/mL of liberase TM (Roche), and 0.125 mmol/L of CaCl_2_ were added while maintaining perfusion until the heart became flaccid. Then, the murine atrium was collected in a perfusion buffer supplemented with 5% bovine calf serum. The tissue was sliced into small pieces and disintegrated by pipetting. Stepwise Ca^2+^ reintroduction was then performed by increasing [Ca^2+^] from 0.1 to 1.0 mmol/L. Owing to the limited number of atrial cardiomyocytes obtained from the cell isolation, only one of the following methods could be performed per subject.

### 2.3 Measurements of atrial ROS production

Isolated atrial cardiomyocytes were plated on laminin-coated recording chambers and loaded with either 5 μmol/L of CellRox™ Orange (Thermo Fisher Scientific) or 5 μmol/L of MitoSox™ Red (Thermo Fisher Scientific) in the presence of 0.04% (w/v) pluronic acid (Invitrogen; 15 min incubation at 37°C). The chambers were then placed on a laser-scanning confocal microscope (Zeiss LSM 700), and measurements were performed in Tyrode’s solution containing 140 mmol/L of NaCl, 4 mmol/L of KCl, 5 mmol/L of HEPES, 1 mmol/L of MgCl_2_, 10 mmol/L of glucose, and 1 mmol/L of CaCl_2_ (pH 7.4 at room temperature with NaOH). The frame scans (CellRox™ Orange: 555 nm excitation, LP 560 nm emission; MitoSox™ Red: 488 nm excitation, LP 490 nm emission) were acquired once every minute for 10 min upon electrical field stimulation (1 Hz). The CellRox™ Orange and MitoSox™ Red fluorescence (F) values were then normalized with respect to the background fluorescence (F/F_0_). The slope of increase in F/F_0_ over time was used as the measure of cellular (CellRox™ Orange) and mitochondrial (MitoSox™ Red) ROS productions.

### 2.4 Epifluorescence microscopy

Intracellular Na^+^ was determined by epifluorescence microscopy using the Na^+^-sensitive sodium-binding benzofuran isophthalate-AM (SBFI-AM) dye (Thermo Fisher Scientific). The isolated atrial cardiomyocytes were plated on laminin-coated measurement chambers and loaded with 10 μmol/L of SBFI-AM for 90 min at room temperature according to manufacturer instructions. The loaded chambers were then placed on the stage of an inverted microscope (Nikon Eclipse TE2000-U) and superfused with Tyrode’s solution containing 140 mmol/L of NaCl, 4 mmol/L of KCl, 5 mmol/L of HEPES, 1 mmol/L of MgCl_2_, 10 mmol/L of glucose, and 1 mmol/L of CaCl_2_ (pH 7.4 at 37°C with NaOH). Regular electrical stimulation was then performed by field stimulation (1, 2, and 4 Hz with 20 V for 4 ms) in a sequential manner for 5 min per frequency. The emissions were obtained using a fluorescence detection system (IonOptix), and the SBFI fluorescence emission ratio was measured by alternating excitations at 340 nm and 380 nm. Then, steady-state measurements averaged over 10 s with ongoing stimulation were analyzed. For some experiments, calibration of the F_340 nm/380 nm_ fluorescence ratio for fixed Na^+^ concentrations (0, 10, and 20 mmol/L) was performed. To achieve this, a K^+^-free solution containing 30 mmol/L of NaCl, 115 mmol/L of Na-gluconate, 10 mmol/L of HEPES, 2 mmol/L of EGTA, and 10 mmol/L of glucose (pH 7.2 at 37°C with TRIS) was mixed with an Na^+^-free solution containing 30 mmol/L of KCl, 115 mmol/L of K-gluconate, 10 mmol/L of HEPES, 2 mmol/L of EGTA, and 10 mmol/L of glucose (pH 7.2 at 37°C with TRIS) in an appropriate proportion to achieve the desired Na^+^ concentration. For all Na^+^ calibration solutions, the ionophore Gramicidin D (10 μmol/L, Sigma-Aldrich) was added to achieve cell permeabilization. For the 10 and 20 mmol/L Na^+^ calibration solutions, an additional 100 μmol/L of the Na^+^/K^+^-ATPase inhibitor strophanthidin (Sigma-Aldrich) was added. Continuous electrical stimulation was then performed at 1 Hz as described above, and the steady-state fluorescence ratio was recorded after 20 min for each step in the calibration process (with Tyrode’s solution for 0, 10, and 20 mmol/L of Na^+^).

The spontaneous Ca^2+^ release events were analyzed by epifluorescence microscopy as described previously (Hegner et al., 2023). In short, the atrial cardiomyocytes were loaded with the Ca^2+^-sensitive dye Fura-2-AM (5 μmol/L, Thermo Fisher Scientific) and subjected to regular electrical field stimulation at 1, 2, and 4 Hz for 5 min per frequency. Deviations from the diastolic Ca^2+^ baseline between two stimulated transients were defined as the spontaneous Ca^2+^ release events and counted by one investigator blinded to the genotype and intervention.

### 2.5 Statistical analysis

The experiments were performed and analyzed after being blinded to the genotype (wild-type vs CaMKIIδ^−/−^) and treatment (CTRL vs PTFE) of the mice, and the results were presented as mean values per mouse ±standard error of the mean (SEM) for three significant digits. The normal distribution was assessed via the Shapiro–Wilk normality test, and student’s t-test was used to compare two normally distributed continuous variables. One-way ANOVA with Holm–Sidak’s *post hoc* correction was performed for comparisons of more than two normally distributed groups. GraphPad PRISM 10 was used to test for differences between the linear regression slopes. Two-sided *p*-values below 0.05 were considered to be statistically significant.

## 3 Results

### 3.1 ROS production is increased in atrial cardiomyocytes of OSA mice

Previously, we demonstrated increased ROS production in the myocardium of patients with SDB (Arzt et al., 2022). Additionally, we were able to show increased ROS production in the ventricular cardiomyocytes of the PTFE-treated mice (Hegner et al., 2023). Since high-risk cardiovascular patients often have various comorbidities, such as diabetes, heart failure, and coronary artery disease, it is difficult to determine the independent effect of SDB on ROS production. Therefore, in this study, we analyzed the effect of specific OSA induction by PTFE treatment in mouse atrial cardiomyocytes.

**FIGURE 2:**
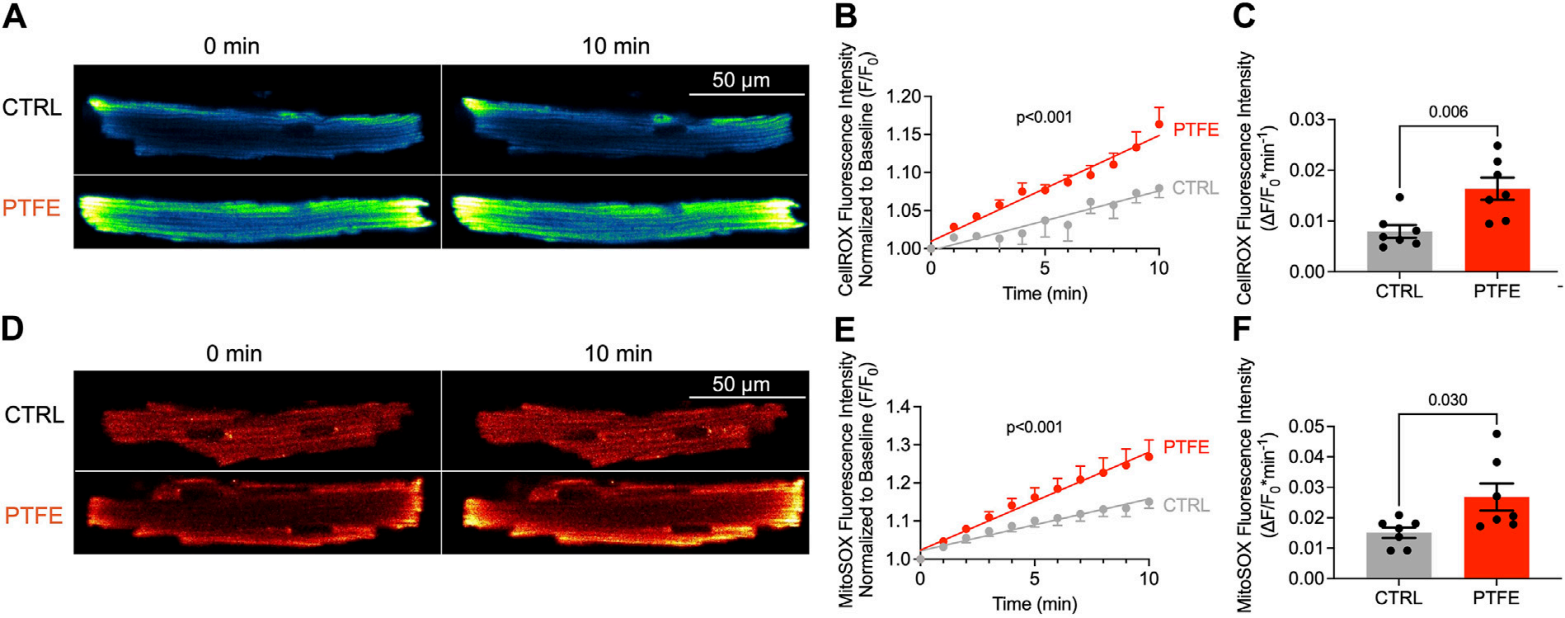
ROS production is increased in the atrial cardiomyocytes of PTFE mice: **(A)** original laser-scanning confocal microscopy images of atrial cardiomyocytes loaded with the CellRox™ Orange dye (artificial coloring of monochrome image with Blue_Yellow LUT); **(B)** linear regression analysis of the cytosolic ROS production over time (n = 15/7 control (CTRL) vs n = 14/7 PTFE); **(C)** mean slope of cytosolic ROS production over time (n = 15/7 CTRL vs n = 14/7 PTFE); **(D)** original laser scanning confocal microscopy images of atrial cardiomyocytes loaded with the MitoSox™ Red dye (artificial coloring of monochrome image with Red_Hot LUT); **(E)** linear regression analysis of the mitochondrial ROS production over time (n = 15/7 CTRL vs n = 13/7 PTFE); **(F)** mean slope of mitochondrial ROS production over time (n = 15/7 CTRL vs n = 13/7 PTFE). N indicates the number of cells/number of mice. The comparisons are based on student’s t-test and linear regression analysis as appropriate.

Eight weeks after the PTFE injections, the cytosolic ROS production in the experimental mice increased compared to those of the control animals (1.63e-2 ± 2.2e-3 in PTFE vs 7.95e-3 ± 1.3e-3 (ΔF/F_0_*min^−1^) in control, *p* = 0.006, n = 7 vs 7, Figures 2A–C). Moreover, the time-dependent cytosolic ROS production estimated by linear regression analysis was elevated in the PTFE-treated mice compared to the controls (r^2^ = 0.666, *p* < 0.001, n = 7 in PTFE vs r^2^ = 0.327, *p* < 0.001, n = 7 in control, and *p* < 0.001 for difference in slopes, Figure 2B).

Similarly, mitochondrial ROS production quantified by MitoSox™ Red was higher in the PTFE-treated mice than the controls (2.68e-2 ± 4.4e-3 in PTFE vs 1.51e-2 ± 1.7e-3 in control, *p* = 0.030, n = 7 vs 7, Figures 2D–F). Congruently, the time-dependent mitochondrial ROS production estimated by linear regression analysis was elevated in the PTFE mice compared to the controls (r^2^ = 0.578, *p* < 0.001, n = 7 in PTFE vs r^2^ = 0.540, *p* < 0.001, n = 7 in control, *p* < 0.001 for difference in slopes, Figure 2E).

### 3.2 CaMKII-dependent dysregulation of atrial Na^+^ homeostasis

**FIGURE 3:**
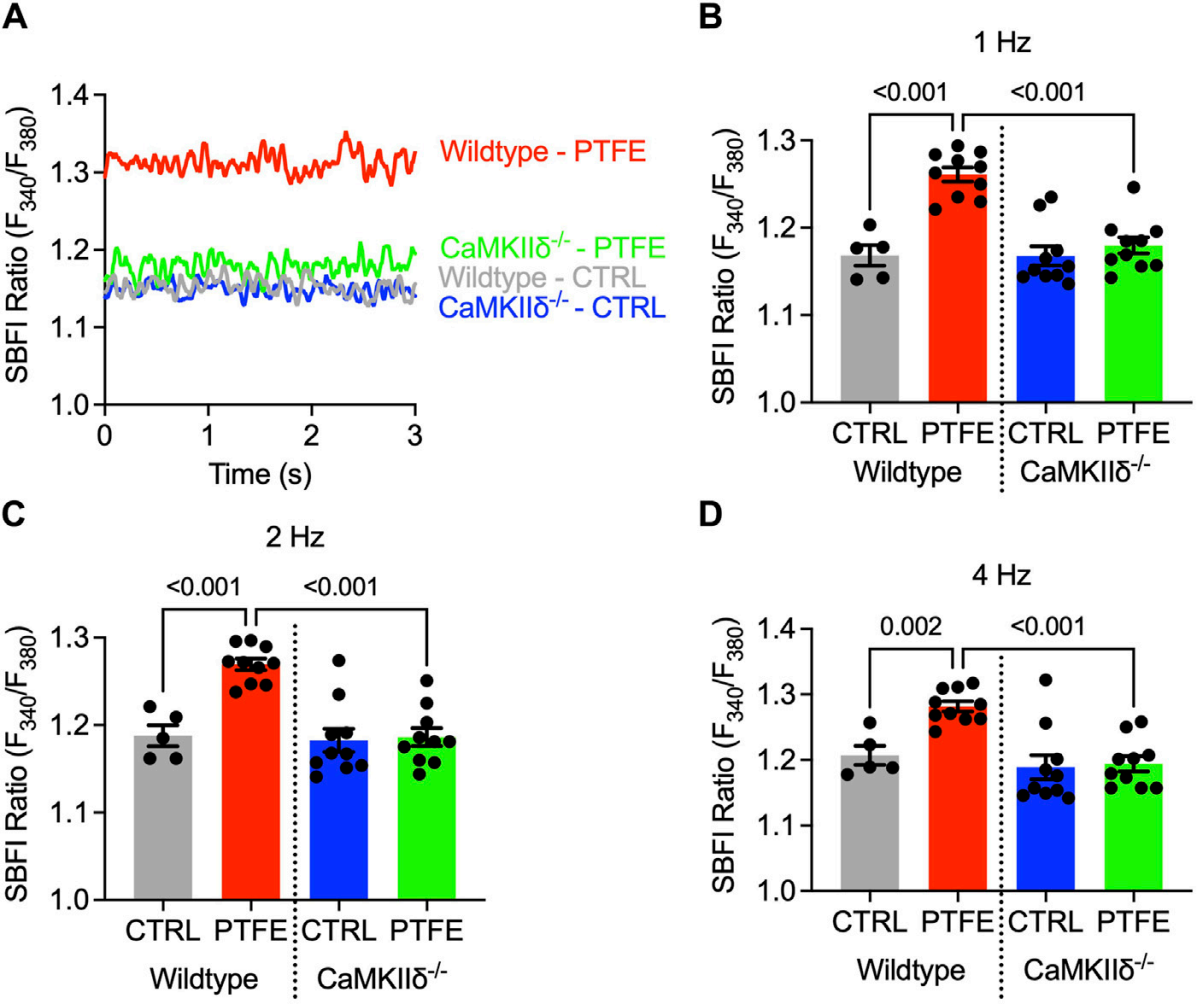
Cytosolic Na^+^ is elevated only in the atrial cardiomyocytes of wild-type PTFE mice: **(A)** original traces of the SBFI ratio (F_340_/F_380_) in the atrial cardiomyocytes; mean SBFI ratios at **(B)** 1 Hz, **(C)** 2 Hz, and **(D)** 4 Hz electrical stimulation (n = 19/5 wild-type control (CTRL), n = 38/10 wild-type PTFE, n = 32/10 CaMKIIδ^−/−^ CTRL, and n = 36/10 CaMKIIδ^−/−^ PTFE). N indicates the number of cells/number of mice. The comparisons are based on one-way ANOVA with Holm–Sidak’s *post hoc* correction.

The atrial cardiomyocyte Na^+^ concentration was assessed by epifluorescence microscopy using the Na^+^-sensitive SBFI-AM fluorescence dye. The cardiomyocytes underwent continuous electrical stimulation at 1, 2, and 4 Hz to account for differences between the physiological human and murine heart rates. The SBFI F_340_/_380_ ratio was analyzed at steady-state levels (Figure 3A). In the wild-type PTFE mice, the SBFI ratio increased to 1.26 ± 8.2e-3 as compared to 1.17 ± 1.2e-2 in the control mice (*p* < 0.001, Figure 3B), while the CaMKIIδ^−/−^ PTFE mice remained protected (*p* < 0.001, Figure 3B). Importantly, the SBFI F_340_/_380_ ratio increased similarly across all frequencies, including 2 and 4 Hz, in the wild-type PTFE mice while remaining at healthy control levels in the CaMKIIδ^−/−^ PTFE mice (Figures 3C, D).

**FIGURE 4:**
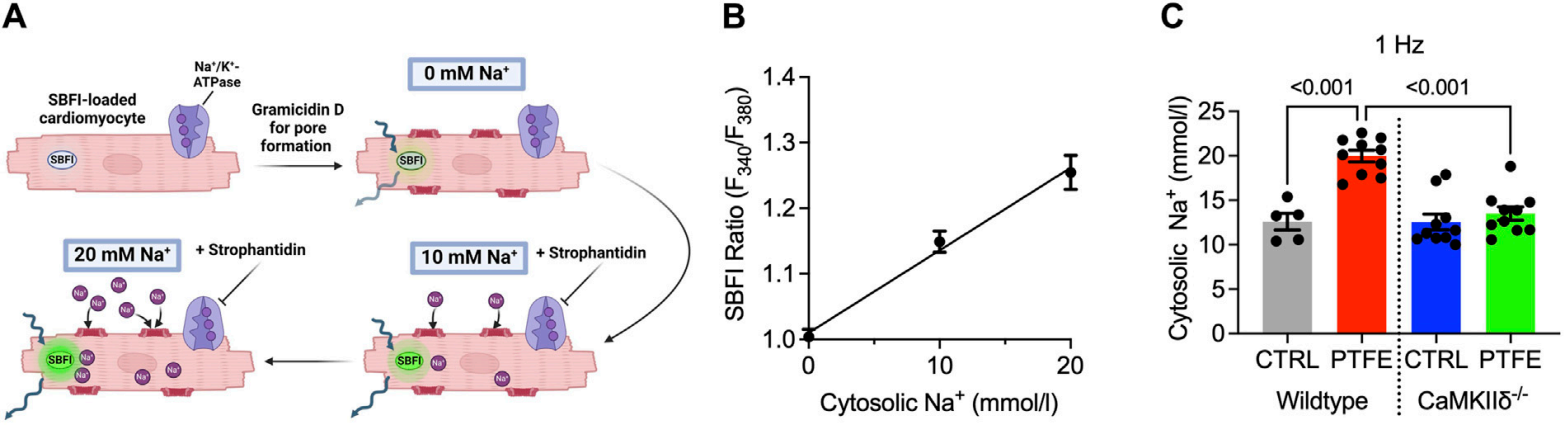
Measurement of Na^+^ concentration and calibration procedure: **(A)** protocol for SBFI-AM calibration to Na^+^ concentration performed in the atrial cardiomyocytes; **(B)** mean SBFI ratios (F_340_/F_380_) at 0, 10, and 20 mmol/L of Na^+^ with linear regression (n = 14 cells); mean intracellular Na^+^ concentration at **(C)** 1 Hz electrical stimulation (n = 19/5 wild-type control (CTRL), n = 38/10 wild-type PTFE, n = 32/10 CaMKIIδ^−/−^ CTRL, and n = 36/10 CaMKIIδ^−/−^ PTFE). N indicates the number of cells/number of mice. The comparisons are based on one-way ANOVA with Holm–Sidak’s *post hoc* correction or linear regression analysis as appropriate.

Calibration experiments were conducted to convert the ratiometric SBFI fluorescence values to Na^+^ concentrations (mmol/L) (Figure 4A). The SBFI fluorescence ratios were plotted for fixed Na^+^ concentrations (0, 10, and 20 mmol/L, Figure 4B). The SBFI F_340_/_380_ ratio was converted to intracellular Na^+^ concentration (mmol/L) using the resulting calibration curve. The atrial cardiomyocyte Na^+^ concentration at 1 Hz increased in the wild-type PTFE mice to 20.0 ± 0.65 from 12.6 ± 0.94 mmol/L in the wild-type control (*p* < 0.001, Figure 4C) but remained at the control level (13.5 ± 0.74 mmol/L) in the CaMKIIδ^−/−^ PTFE mice (*p* < 0.001 vs wild-type PTFE, Figure 4C). At 2 Hz stimulation, the Na^+^ concentration increased to 20.6 ± 0.53 mmol/L in the wild-type PTFE mice from 14.1 ± 0.96 mmol/L in the wild-type control (*p* < 0.001). During 4 Hz stimulation, the intracellular Na^+^ concentration increased further to 21.6 ± 0.62 mmol/L in the wild-type PTFE mice from 15.7 ± 1.2 mmol/L in the control (*p* = 0.002). Moreover, at 2 and 4 Hz, Na^+^ concentrations in the CaMKIIδ^−/−^ PTFE mice were similar to those of the wild-type control mice (2 Hz: 14.0 ± 0.82 mmol/L, *p* < 0.001 vs wild-type PTFE; 4 Hz: 14.6 ± 0.93 mmol/L, *p* < 0.001 vs wild-type PTFE).

### 3.3 CaMKII-dependent arrhythmias in isolated atrial myocytes of OSA mice

**FIGURE 5:**
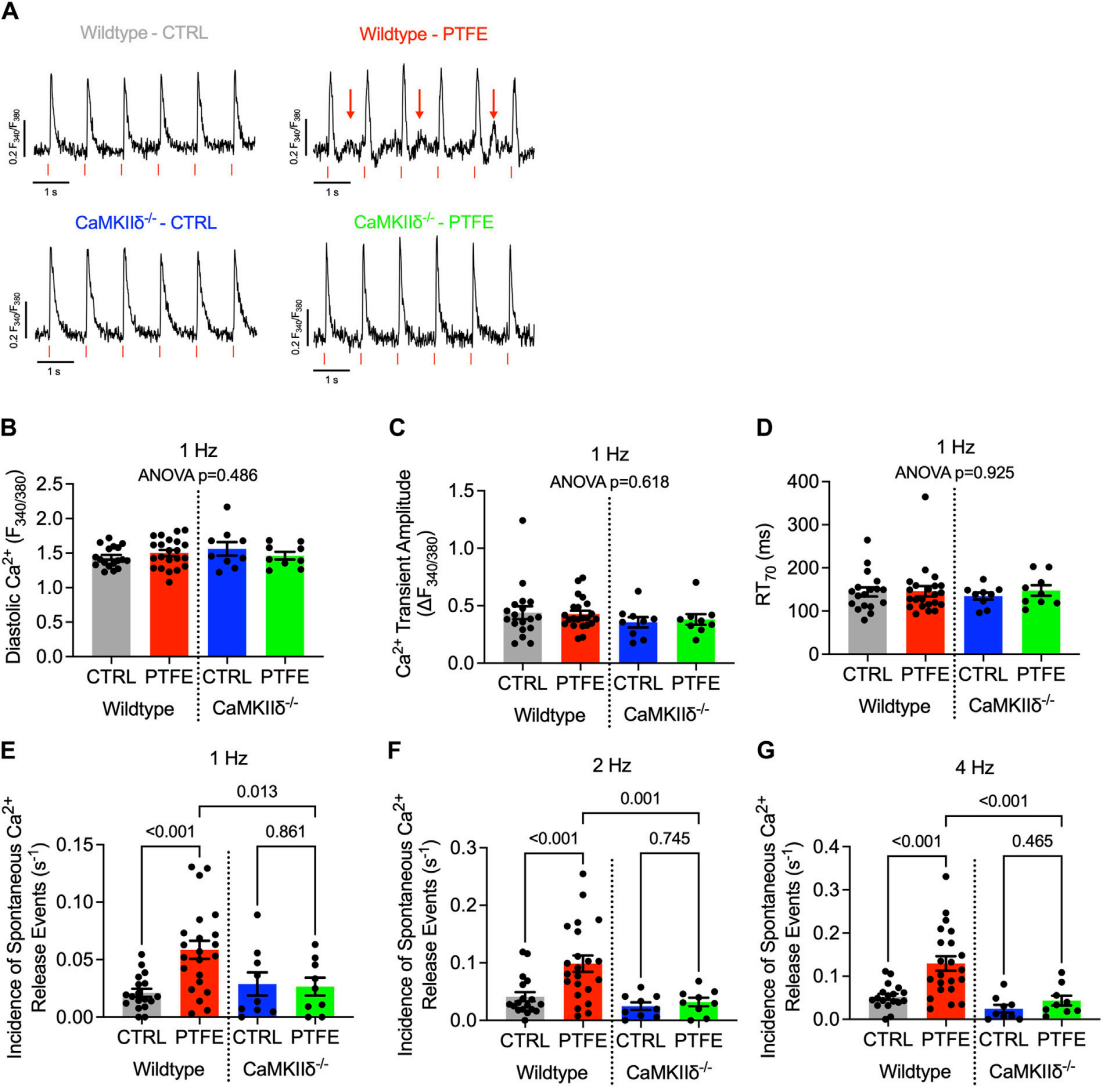
CaMKIIδ^−/−^ mice are protected from spontaneous Ca^2+^ release events: **(A)** original recordings of Ca^2+^ transients (Fura-2 ratio, F_340_/F_380_) in the atrial cardiomyocytes, where the spontaneous Ca^2+^ release events are indicated by red arrows in the wild-type PTFE mice; **(B)** mean diastolic Ca^2+^, **(C)** Ca^2+^ transient amplitude, and **(D)** relaxation time to 70% of baseline at 1 Hz with ANOVA; *p* = n.s. Incidence of spontaneous Ca^2+^ release events at **(E)** 1 Hz, **(F)** 2 Hz, and **(G)** 4 Hz electrical stimulation. N = 57/18 wild-type control (CTRL), n = 66/22 wild-type PTFE, n = 27/9 CaMKIIδ^−/−^ CTRL, and n = 26/9 CaMKIIδ^−/−^ PTFE. N indicates the number of cells/number of mice. The comparisons are based on one-way ANOVA with Holm–Sidak’s *post hoc* correction.

Spontaneous Ca^2+^ release events were assessed in isolated atrial cardiomyocytes loaded with the Ca^2+^-sensitive Fura-2-AM dye during regular electrical stimulation. Non-stimulated pro-arrhythmic events could be observed in the myocytes from the wild-type PTFE mice (Figure 5A, indicated by red arrows), while the Ca^2+^ transient characteristics remained unaltered in the PTFE mice (Figures 5B–D). At 1 Hz stimulation, the incidence of spontaneous Ca^2+^ release events increased in the wild-type PTFE mice by more than two-fold to 5.85e-2 ± 7.9e-3 (s^−1^) from 2.11e-2 ± 3.5e-3 in the wild-type control mice (*p* < 0.001, Figure 5E). Atrial cardiomyocytes from the CaMKIIδ^−/−^ PTFE mice were protected from such an increase in the rate of arrhythmias (2.65e-2 ± 7.8e-3, *p* = 0.007 vs wild-type PTFE, Figure 5E). Similar effects were also observed at 2 Hz stimulation, with the rate of pro-arrhythmic non-stimulated events increasing to 9.86e-2 ± 1.4e-2 in the wild-type PTFE mice from 4.11e-2 ± 8.0e-3 in the wild-type control mice (*p* < 0.001, Figure 5F), whilst the CaMKIIδ^−/−^ PTFE mice exhibited no increase in the frequency of spontaneous Ca^2+^ release events (3.20e-2 ± 7.4e-3, *p* < 0.001 vs wild-type PTFE, Figure 5F). At a stimulation rate of 4 Hz, which is closer to the physiological murine heart rate (Li et al., 1999), the rate of atrial pro-arrhythmic events remained elevated by more than two-fold in the wild-type PTFE mice compared to the control (1.29e-1 ± 1.7e-2 vs 5.24e-2 ± 6.8e-3, *p* < 0.001, Figure 5G). Once again, atrial cardiomyocytes from the CaMKIIδ^−/−^ PTFE mice exhibited arrhythmia frequencies comparable to those of the healthy controls (4.34e-2 ± 1.1e-2, *p* < 0.001 vs wild-type PTFE, Figure 5G). Additionally, no significant differences were observed between the CaMKIIδ^−/−^ control and PTFE mice (Figures 5E–G).

## 4 Discussion

In the present study, we show increased ROS production, Na^+^ overload, and more frequent spontaneous Ca^2+^ release events in the atrial cardiomyocytes of OSA mice. The current therapeutic strategies for SDB are mostly limited to lifestyle interventions and CPAP therapy (Aurora et al., 2012; Randerath et al., 2017; Patil et al., 2019). However, patient compliance is often low in such cases, and interventions such as adaptive servo-ventilation therapy may even be detrimental in certain patients (Cowie et al., 2015; McEvoy et al., 2016). Although SDB is associated with increased incidence of atrial fibrillation and lower sustained success of cardioversion or pulmonary vein isolation (Gami et al., 2004; Gami et al., 2007; Linz et al., 2018), CPAP therapy has failed to reduce the arrhythmia burden and incidence of adverse cardiovascular events (Peker et al., 2016; Traaen et al., 2021). Additionally, SDB patients have been reported to frequently suffer from heart failure, especially HFpEF (Lebek et al., 2021; Levy et al., 2022; Wester et al., 2023). These aspects highlight the urgent need for more targeted and effective therapies for SDB patients.

Recently, we showed for the first time that intracellular Na^+^ entry and Na^+^ concentration are higher in the atrial myocytes of patients with HFpEF, a condition in which SDB is very common, which could contribute to atrial contractile dysfunction and arrhythmias (Trum et al., 2024). Interestingly, we also showed that patients with SDB have increased late Na^+^ current in their remodeled atria, which could contribute to intracellular Na^+^ overload (Lebek et al., 2022). However, because these patients could also have various comorbidities, it is very difficult to determine the standalone effects of OSA.

The SDB mouse model utilized in this study is ideal for exploration of the pathological mechanisms and novel therapeutic targets as it is devoid of the confounding comorbidities frequently exhibited by patients; the mouse model is also more widely available than SDB patient biomaterial (Lebek et al., 2020a; Hegner et al., 2023). It is noted that these mice developed diastolic and mild systolic left-ventricular dysfunctions, which also resulted in increased heart and lung weights (Lebek et al., 2020a; Hegner et al., 2023). It is therefore possible that the effects observed in the ventricles may contribute to changes in the atria.

### 4.1 SDB-dependent pathological mechanisms promoting arrhythmias

The frequently discussed pro-arrhythmic mechanisms that could facilitate atrial fibrillation in SDB include intrathoracic pressure changes (Linz et al., 2011), autonomous imbalance and beta-adrenergic stimulation during nocturnal awakening periods (Abboud and Kumar, 2014), increased arterial blood pressure (Hetzenecker et al., 2013), structural remodeling (Anter et al., 2017), conduction abnormalities (Anter et al., 2017; Hegner et al., 2021b), ion-channel dysfunction and triggered activity (Lebek et al., 2020b; Lebek et al., 2022), and intermittent hypoxia/desaturation (Tkacova et al., 1998; Iwasaki et al., 2014). The latter is also a strong inductor of oxidative stress and ROS production (Gozal and Kheirandish-Gozal, 2008). Indeed, we previously observed increased production of cytosolic ROS in human atrial tissues of SDB patients (Lebek et al., 2020b). In agreement with these observations, in this study, we report increased cytosolic and mitochondrial ROS production in the atrial myocytes of OSA mice without comorbidities.

ROS have been shown to oxidize many ion channels and transporters. Indeed, direct oxidation of the ryanodine type-2 receptors (RyR2) can promote increased diastolic sarcoplasmic reticulum Ca^2+^ release and subsequent arrhythmias (Huang et al., 2021). On the other hand, CaMKIIδ is a kinase central to myocardial Na^+^ and Ca^2+^ homeostasis that can also be directly oxidized at methionine-281 and -282, thereby releasing the catalytic domain leading to increased enzyme activation (Erickson et al., 2008; Lebek et al., 2023b; Lebek et al., 2024).

Our group previously established that cardiac CaMKIIδ activity is pathologically increased in SDB patients and also in SDB mice in the model used in this study (Lebek et al., 2020a; Lebek et al., 2020b; Arzt et al., 2022; Hegner et al., 2023). In the present study, we present data from isolated atrial cardiomyocytes, but the limited amount of tissue precluded further protein target analysis, which is a potential limitation of this study. There are several important downstream targets of CaMKIIδ, including voltage-gated Na^+^ channels Na_V_1.5 and Na_V_1.8, RyR2, phospholamban, L-type Ca^2+^ channels, and Na^+^/Ca^2+^ exchangers, which have been shown to be involved in arrhythmogenesis (Bers, 2002; Fischer et al., 2013; Bengel et al., 2021). CaMKIIδ overactivation in SDB can lead to disturbed Ca^2+^ homeostasis, including increased sarcoplasmic reticulum Ca^2+^ leakage, pro-arrhythmic non-stimulated events in humans and mice, and multicellular arrhythmias in the patient trabeculae (Lebek et al., 2020b; Arzt et al., 2022; Hegner et al., 2023). These pro-arrhythmic events could serve as triggers of atrial fibrillation (Nattel et al., 2020).

### 4.2 Disturbance of atrial Na^+^ homeostasis as a novel pathological mechanism in SDB

Increased CaMKIIδ activation can facilitate intracellular Na^+^ level overload (Wagner et al., 2006; Wagner et al., 2011), and recent studies have highlighted the interactions between CaMKIIδ and increased Na^+^ influx in heart failure (Bengel et al., 2021), resulting in increased myocyte Na^+^ concentration (Despa, 2018). One of the proposed mechanisms is increased late Na^+^ current (late I_Na_), which was detected in the atrial myocytes of patients with SDB (Lebek et al., 2020b; Lebek et al., 2022). However, data regarding Na^+^ in the mouse atrial myocytes is scarce as the biomaterial is limited by the small murine atrium and methodological challenges (Garber et al., 2022). Garber et al. (2017, 2022) recommend calibrating each myocyte individually, which we did not perform for every cell in this study with the aim of increasing the yield. Consequently, the converted Na^+^ concentrations may be more general estimates. The quiescent murine atrial myocyte Na^+^ concentrations were previously reported at ∼8 mmol/L with an increase to 11–12 mmol/L at 1 Hz stimulation. Since the Na^+^ concentration increases in a frequency-dependent manner (Despa et al., 2002; Pieske et al., 2002), we conducted measurements at multiple frequencies (1, 2, and 4 Hz) to account for the increased rates that are commonly seen in human atrial arrhythmias (Lu and Chen, 2021). In addition, this allowed us to take into account the physiologically different heart rates of humans and mice to offer a more comprehensive translational perspective. Our data are in direct agreement with the findings of previously published literature as we estimated the atrial myocyte Na^+^ concentration to be ∼12 mmol/L at 1 Hz stimulation in healthy wild-type mice.

Importantly, at all the tested frequencies, the Na^+^ concentrations in the atrial cardiomyocytes were profoundly higher in the OSA mice in excess of Δ+5 mmol/L. Owing to the selected calibration range of 0–20 mmol/L Na^+^ (Figure 4B), any reported concentrations above 20 mmol/L may even be underestimated. An increase in the intracellular Na^+^ by this margin impairs Na^+^/Ca^2+^ exchanger (NCX) function owing to reduced transmembrane Na^+^ gradients in a manner similar to that observed in heart failure (Despa et al., 2002; Pieske et al., 2002; Hegner et al., 2022). Impaired NCX function may further increase the cellular Ca^2+^ levels by reduced Ca^2+^ export, which could further increase CaMKIIδ activation in a Ca^2+^-dependent fashion, thereby exacerbating Na^+^ increase (Sapia et al., 2010; Bengel et al., 2021). Moreover, increased Na^+^ influx is linked to initiation of atrial fibrillation (Sossalla et al., 2010; Wan et al., 2016). Cellular Na^+^ overload is also known to increase cytosolic and mitochondrial ROS productions (Kohlhaas et al., 2010). Indeed, we measured increased intracellular and mitochondrial ROS productions in the cardiomyocytes of OSA mice. In turn, this could promote a vicious cycle by leading to further Na^+^ increase via CaMKIIδ activation. Importantly, we did not observe any increase in atrial Na^+^ concentrations in the cardiomyocytes of CaMKIIδ^−/−^ SDB mice at any of the evaluated frequencies.

In line with the disturbed Na^+^ homeostasis, we also observed more than two-fold increase in pro-arrhythmic events in the atrial cardiomyocytes of the wild-type SDB mice at all stimulation frequencies (1, 2, and 4 Hz), which was almost similar to the levels of healthy controls in the CaMKIIδ^−/−^ SDB mice. Moreover, production of ROS has been linked to arrhythmogenesis in cardiomyocytes (Liu et al., 2022). Importantly, ROS production and NADPH oxidase activity are higher in SDB (Gozal and Kheirandish-Gozal, 2008), whereas the other Ca^2+^ transient characteristics remain unaltered in the PTFE mice. This may be attributed to compensatory effects on the sarcoplasmic reticulum Ca^2+^ content, as observed in patients with paroxysmal atrial fibrillation (Voigt et al., 2014). We previously reported a reduced Ca^2+^ transient amplitude in the ventricular cardiomyocytes of SDB mice (Hegner et al., 2023), which we did not observe in the atrial cardiomyocytes in the present study.

Our data suggest that modulation of CaMKIIδ activity could be a promising antiarrhythmic approach in SDB. Even as pharmacological inhibition of CaMKIIδ is being investigated (Pellicena and Schulman, 2014; Lebek et al., 2018), CRISPR-Cas9 gene editing of *CAMK2D* could be an advanced strategy to overcome the previous limitations, as this technology has been used with >2,000-fold increased specificity toward *CAMK2D* compared to other isoforms (Lebek et al., 2023a). Additionally, pharmacological inhibition and genetic ablation of (oxidative) activation of CaMKIIδ have been shown to protect from pro-arrhythmic activities (Lebek et al., 2018; Lebek et al., 2023a; Lebek et al., 2023b; Hegner et al., 2023).

## 5 Conclusion

Patients with SDB are at increased risk of developing atrial fibrillation and have demonstrated lower efficacy for currently available anti-arrhythmic therapies. In fact, targeted anti-arrhythmic therapies are completely lacking in SDB. In the present study, we demonstrated that in an SDB mouse model devoid of comorbidities, the production of cytosolic and mitochondrial ROS increased in the atrial cardiomyocytes. ROS are known to facilitate persistent overactivation of Ca^2+^/calmodulin-dependent protein kinase IIδ (CaMKIIδ), which results in disruption of the cellular Na^+^ and Ca^2+^ homeostasis. Herein, we describe elevated Na^+^ concentrations at multiple stimulation frequencies associated with higher chances of spontaneous Ca^2+^ release events in SDB mice. Importantly, the CaMKIIδ^−/−^ mice were protected from such effects. Therefore, inhibition of CaMKIIδ in SDB may reduce Na^+^ overload and protect against arrhythmias, which could have therapeutic implications in the future.

## Data Availability

The original contributions presented in the study are included in the article/Supplementary material; further inquiries can be directed to the corresponding authors.
